# Antiviral Therapy in Lamivudine-Resistant Chronic Hepatitis B Patients: A Systematic Review and Network Meta-Analysis

**DOI:** 10.1155/2016/3435965

**Published:** 2016-09-08

**Authors:** Hui-Lian Wang, Xi Lu, Xudong Yang, Nan Xu

**Affiliations:** ^1^Department of Genetics and Molecular Biology, Xi'an Jiaotong University School of Medicine, Xi'an, Shaanxi 710061, China; ^2^Key Laboratory of Environment and Genes Related to Diseases (Xi'an Jiaotong University), Ministry of Education, Xi'an, Shaanxi 710061, China; ^3^School of Mechanical Engineering, Xi'an Jiaotong University, Xi'an, Shaanxi 710049, China; ^4^2nd Affiliated Hospital, Xi'an Jiaotong University School of Medicine, Xi'an, Shaanxi 710004, China

## Abstract

The relative efficacy of different strategies for chronic hepatitis B (CHB) patients with lamivudine resistance (LAM-R) has not yet been systematically studied. Clinical trials were searched in PUBMED, MEDLINE, EMBASE, and CNKI databases up to February 15, 2016. Nine trials including 764 patients met the entry criteria. In direct meta-analysis, TDF showed a stronger antiviral effect than any one of ETV, LAM/ADV, and ADV against LAM-R hepatitis B virus. LAM/ADV therapy was superior to ADV in suppressing viral replication. ETV achieved similar rate of HBV DNA undetectable compared to ADV or LAM/ADV. In network meta-analysis, TDF had higher rates of HBV DNA undetectable compared to ETV (OR, 24.69; 95% CrI: 5.36–113.66), ADV (OR, 37.28; 95% CrI: 9.73–142.92), or LAM/ADV (OR, 21.05; 95% CrI: 5.70–77.80). However, among ETV, ADV, and LAM/ADV, no drug was clearly superior to others in HBV DNA undetectable rate. Moreover, no significant difference in the rate of ALT normalization or HBeAg loss was observed compared the four rescue strategies with each other. TDF appears to be a more effective rescue therapy than LAM/ADV, ETV, or ADV. LAM plus ADV therapy was a better treatment option than ETV or ADV alone for patients with LAM-R.

## 1. Introduction

Chronic hepatitis B virus (HBV) infection remains a major public health problem and affects approximately 400 million people worldwide, especially in Asia [1]. Large cohort studies have demonstrated that the risk of liver disease progression in patients with chronic hepatitis B (CHB) is associated with elevated HBV DNA levels [2]. Therefore, the goals of therapy in HBV infected patients are to reverse progression of the disease by long-term suppression of HBV replication [3]. With the availability of potent nucleos(t)ide analogues (NAs), such as tenofovir disoproxil fumarate (TDF) and entecavir (ETV), suppression of serum HBV DNA to undetectable levels is achievable in most NA-naive patients in the absence of drug-resistant HBV mutants [4, 5]. However, for most NAs such as lamivudine (LAM) and adefovir (ADV), one of the major limitations of these drugs is resistance development in long-term treatment, which can lead to a rebound in HBV replication and exacerbation of HBV-related disease [2].

LAM was prescribed extensively for the treatment of CHB in the era of the early generation NAs. However, resistance to LAM emerges in approximately 20% of patients after 1 year and in 70% of patients after 5 years of treatment [6]. Until TDF rescue therapy becomes available, switching to ADV or ETV monotherapy and adding on ADV were once suggested against LAM-R HBV in earlier international guidelines [3, 7]. Unfortunately, sequential ADV monorescue therapy for LAM-R induced high resistance to ADV [8, 9], and ETV monorescue therapy resulted in 50% of these patients developing ETV-resistance (ETV-R) after 5 years of treatment [10]. Combination of ADV and LAM therapy reduces the development of ADV resistance and has been a practical option for treatment of LAM resistance. However, LAM plus ADV therapy has limited antiviral efficacy in LAM-R patients, and a substantial proportion of patients show a suboptimal virological response which may then result in selection of multidrug-resistant HBV variants and progression of liver disease [11]. As rescue therapies for LAM-R patients, TDF, an oral NA with the most potent activity against HBV and high genetic barrier [12], has demonstrated favourable virological outcomes. Some recent studies have revealed that TDF monotherapy was highly effective in LAM-R patients as well as NA-naïve patients, and the presence of resistance mutations to LAM did not alter the response rates [13, 14].

Although some meta-analyses [24, 25] have compared the efficacy of LAM/ADV versus ETV or ADV for patients with LAM-R, the relative efficacy of various rescue strategies in LAM-R patients has not yet been systematically studied, as well as the efficacy of TDF versus LAM/ADV or ETV or ADV. Network meta-analysis can help assess comparative effectiveness of multiple interventions and synthesize evidence through simultaneous analysis of direct evidence and indirect evidence to calculate a mixed-effect estimate as the weighted average of the two. Such a technique may improve the precision of the estimate (compared with direct evidence alone) and also allows estimation of the comparative efficacy of two active treatments, even if no studies directly compare them [26]. Therefore, in this study, we conducted a network meta-analysis with updated evidence to evaluate effects of different rescue strategies including TDF, ETV, LAM/ADV, and ADV in the treatment of LAM-R patients.

## 2. Materials and Methods

### 2.1. Search Strategy

We searched PUBMED, MEDLINE, EMBASE, and CNKI databases up to February 15, 2016. The following keywords were used for the search to find relevant citations: chronic hepatitis B, lamivudine-resistant, tenofovir, adefovir, and entecavir. In addition, reference lists from retrieved documents were reviewed, and a manual search was conducted to supplement the computer search. The search results were downloaded to a reference database and were further screened by 2 authors (Hui-Lian Wang and Xudong Yang).

### 2.2. Inclusion and Exclusion Criteria

The following inclusion criteria were used for this meta-analysis: (1) CHB patients with LAM resistance which was defined as the presence of HBV variants with amino acid substitutions conferring resistance against LAM (rtM204V/I ± rtL180M); (2) intervention therapies: TDF, ETV or ADV, or LAM plus ADV therapy. The following types of studies were excluded: (1) studies of patients with liver failure, HCC, coinfection with hepatitis C, hepatitis D or HIV, and previous liver transplant, (2) studies not reporting any efficacy measures or not conveying sufficient statistical information, and (3) studies that were not controlled trials.

### 2.3. Efficacy Measures

Efficacy was assessed by rates of undetectable HBV DNA (<400 copies/mL), ALT normalization (<40 IU/mL), HBeAg loss, and virological breakthrough for patients 24, 48, and 96 weeks after therapy.

### 2.4. Data Extraction

Data extraction was assessed independently by two reviewers (Hui-Lian Wang and Xudong Yang). Discrepancies among reviewers were resolved by discussions between the reviewers or by a third person (Nan Xu). Basic information obtained from each eligible trial included the study design, patient characteristics, numbers in each group, and treatments. Data were reviewed to eliminate duplicate reports of the same trial.

### 2.5. Quality Assessment

The risk of bias of included trials was assessed by Cochrane Collaboration's tool with the outcome shown in Sup. Figure 2 in Supplementary Material available online at http://dx.doi.org/10.1155/2016/3435965. The following factors were taken into consideration for the risk of bias: random sequence generation (selection bias), allocation concealment (selection bias), blinding of participants and personnel (performance bias), blinding of outcome assessment (detection bias), incomplete outcome data (attrition bias), selective reporting (reporting bias), and other bias. The risk of bias of each study was explicitly determined on those factors and classified as three different levels: low, high, or unclear. The percentages of low risk of selection bias, performance bias, and the detection bias were less than 50% according to the description of each study. The percentages of low risk of bias of incomplete outcome data, selective reporting, and other bias were all more than 50%. The outcome of risk of bias graph showed that there was low risk of bias in this meta-analysis.

### 2.6. Statistical Analysis

For direct meta-analysis, the data was conducted on continuous and dichotomous outcomes and assessed by Revman. The *χ* ^2^ and *I* ^2^ tests were first calculated to assess the heterogeneity of the included trials. For *P* values more than 0.1, the fixed-effects model was used because of the homogeneity; otherwise, data need to be dealt with with the random-effects model. Pooled odds ratios (OR) with 95% confidence intervals (95% CI) were calculated using either the fixed-effects model (M-H methods) or random-effects model (D-L methods). A two-tailed *P* value of less than 0.05 suggested statistical significance.

We used network meta-analysis methods to compare LAM-R patients with different incorporating evidence on both direct and indirect comparisons. Network meta-analysis was performed using R version 3.2.2 to calculate point estimates (OR) with 95% credibility intervals (CrI) and generate forest plots using random-effects models comparing the effect estimates of different therapies relative to comparator. Rank probabilities were generated to determine the rank of therapies in which the given treatment ranked first as the most effective therapy, second, and so on.

Network meta-analysis was conducted with R 3.2.2, addis and stata 13.0. Direct meta-analysis and figures of risk of bias were generated through Review Manager 5.3.

## 3. Results

### 3.1. Study Characteristics and Quality Assessment

After the selection procedure (Sup. Figure 1), A total of nine studies [15–23] met the inclusion criteria for this review, including 764 patients with LAM-R. Among nine studies, two studies compared TDF versus LAM/ADV [16, 17] and one study compared TDF versus ETV [16] or ADV [15], respectively, six studies compared ETV versus LAM/ETV [18–22], and two studies compared ADV versus LAM/ADV [22, 23]. All nine studies were published in English and in full-text form. The characteristics of each study are listed in Table 1.

### 3.2. Virological Response

#### 3.2.1. Direct Meta-Analysis

**Figure 1:**
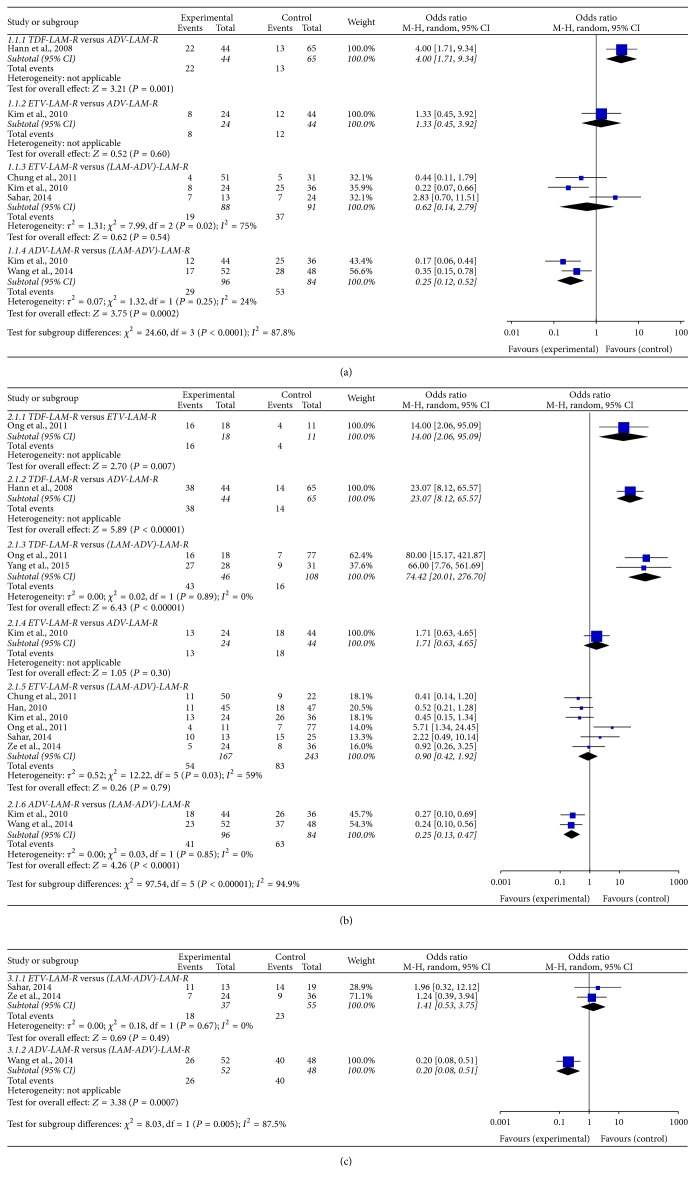
Forest plot of direct meta-analysis of undetectable HBV DNA rate. (a) 24 weeks; (b) 48 weeks; (c) 96 weeks.

At 24 weeks of treatment (Figure 1(a)), TDF had higher HBV DNA undetectable rate compared to ADV (50.0% versus 20.0%; OR, 4.00; 95% CI: 1.71–9.34). ADV had lower HBV DNA undetectable rate compared to LAM/ADV (30.2% versus 63.1%; OR, 0.25; 95% CI: 0.12–0.52). ETV had similar rates of HBV DNA undetectable compared to ADV (33.3% versus 27.3%; OR, 1.33; 95% CI: 0.45–3.92) or LAM/ADV (21.6% versus 40.7%; OR, 0.62; 95% CI: 0.14–2.79), respectively.

At 48 weeks of treatment (Figure 1(b)), TDF had higher rates of HBV DNA undetectable compared to ETV (88.9% versus 36.4%; OR, 14.00; 95% CI: 2.06–95.09), ADV (86.4% versus 21.5%; OR, 23.07; 95% CI: 8.12–65.57), or LAM/ADV (93.5% versus 14.8%; OR, 74.42; 95% CI: 20.01–276.70), respectively. ADV had lower HBV DNA undetectable rate compared to LAM/ADV (42.7% versus 75.0%; OR, 0.25; 95% CI: 0.13–0.47). ETV had similar rates of HBV DNA undetectable compared to ADV (54.2% versus 40.9%; OR, 1.71; 95% CI: 0.63–4.65) or LAM/ADV (32.3% versus 34.2%; OR, 0.90; 95% CI: 0.42–1.92), respectively.

At 96 weeks of treatment (Figure 1(c)), ADV had lower HBV DNA undetectable rate compared to LAM/ADV (50.0% versus 83.3%; OR, 0.20; 95% CI: 0.08–0.51). ETV had similar rates of HBV DNA undetectable compared to LAM/ADV (48.6% versus 41.2%; OR, 1.41; 95% CI: 0.53–3.75).

#### 3.2.2. Network Meta-Analysis

On Bayesian network meta-analysis (Sup. Table 1), TDF had higher rates of HBV DNA undetectable as compared to ETV (OR, 24.67; 95% CrI: 5.36–113.66), ADV (OR, 37.28; 95% CrI: 9.73–142.92), or LAM/ADV (OR, 21.05; 95% CrI: 5.70–77.80) through 48 weeks of treatment. However, among ETV, ADV, and LAM/ADV, no drug was clearly superior to others in HBV DNA undetectable rate during the same period.

TDF, LAM/ADV, and ETV had the highest probability of being ranked first, second, and third for achieving HBV DNA undetectable after 48-week treatment, respectively, whereas ADV had had highest probability of being ranked fourth (Figure 3).

### 3.3. Biochemical Response

#### 3.3.1. Direct Meta-Analysis

After 48-week treatment (Sup. Figure 3), no significant difference in the rate of ALT normalization was observed when comparing TDF to ADV (59.1% versus 69.2%; OR, 0.64; 95% CI: 0.29–1.43) or LAM/ADV (89.3% versus 67.7%; OR, 3.97; 95% CI: 0.96–16.33), respectively. Moreover, there was no significant difference in ALT normalization between ETV and LAM/ADV (77.7% versus 84.0%; OR, 0.75; 95% CI: 0.35–1.58) or ADV and LAM/ADV (71.2% versus 79.2%; OR, 0.65; 95% CI: 0.26–1.63).

#### 3.3.2. Network Meta-Analysis

On Bayesian network meta-analysis, when comparing TDF to others, including ETV, ADV, and LAM/ADV, no drug was clearly superior to others (Sup. Table 2) in the rates of ALT normalization after 48 weeks of therapy.

TDF, ADV, and LAM/ADV had the highest probability of being ranked first, second, and third for improving biochemical response after 48-week treatment, respectively, whereas ETV had had highest probability of being ranked fourth (Sup. Figure 5).

### 3.4. Serological Response

#### 3.4.1. Direct Meta-Analysis

After 48-week treatment (Sup. Figure 4(A)), no significant difference in the rate of HBeAg loss was observed when comparing TDF to ADV (9.1% versus 4.6%; OR, 2.07; 95% CI: 0.44–9.73) or LAM/ADV (3.6% versus 0; OR, 3.44; 95% CI: 0.13–87.85), respectively. Moreover, there was no significant difference in HBeAg loss between ETV and LAM/ADV (3.6% versus 9.4%; OR, 0.41; 95% CI: 0.09–1.89) or ADV and LAM/ADV (48.1% versus 66.7%; OR, 0.46; 95% CI: 0.21–1.04).

After 96-week treatment (Sup. Figure 4(B)), no significant difference in the rate of HBeAg loss was observed when comparing ETV to ADV (33.3% versus 50.0%; OR, 0.50; 95% CI: 0.18–1.41) or LAM/ADV (21.6% versus 32.8%; OR, 0.48; 95% CI: 0.17–1.31), respectively. Moreover, there was no significant difference in HBeAg loss between ADV and LAM/ADV (54.2% versus 64.3%; OR, 0.66; 95% CI: 0.36–1.22).

#### 3.4.2. Network Meta-Analysis

On Bayesian network meta-analysis, when comparing TDF to others, including ETV, ADV, and LAM/ADV, no drug was clearly superior to others (Sup. Table 3) in the rates of HBeAg loss after 48 weeks of therapy.

TDF, LAM/ADV, and ADV had the highest probability of being ranked first, second, and third for achieving HBeAg loss after 48-week treatment, respectively, whereas ETV had highest probability of being ranked fourth (Sup. Figure 6).

### 3.5. Viral Breakthrough and Genotypic Resistance

#### 3.5.1. Direct Meta-Analysis

**Figure 2:**
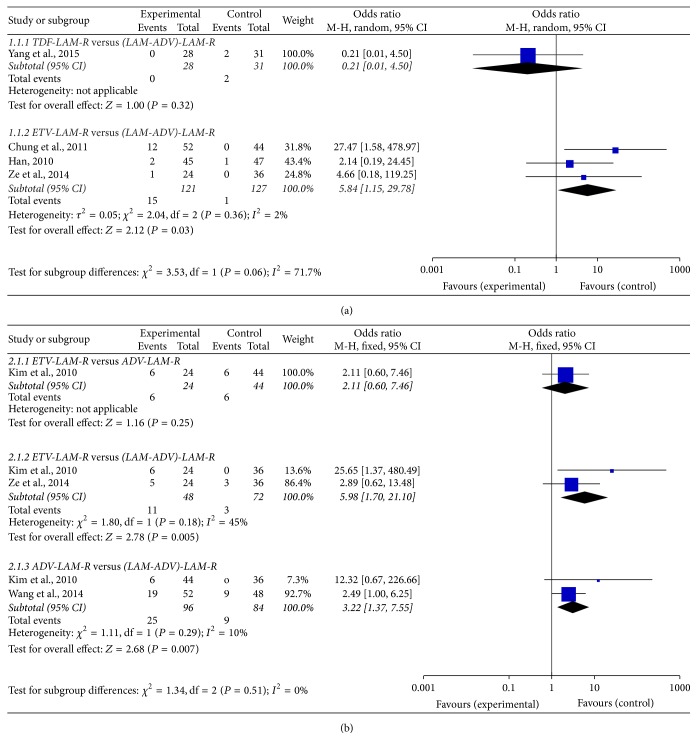
Forest plot of direct meta-analysis of viral breakthrough and genotypic resistance. (a) 48 weeks; (b) 96 weeks.

**Figure 3:**
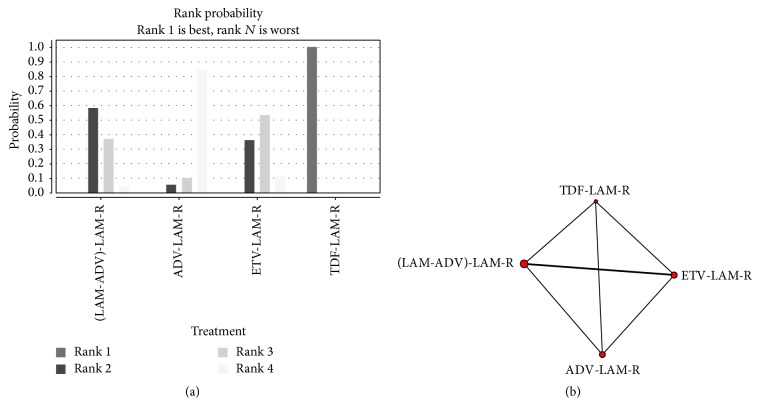
Rank probability and network plot of 48 weeks of undetectable HBV DNA rate. (a) rank probability. Different color indicates different rank showed in the picture; for example, the lightest color means rank 4. The highest bar in the chart of one intervention represents its rank probability. Rank 1 means the best efficiency, and rank 4 means the worst. The bar which means rank 1 in TDF treatment group is the highest bar; therefore, TDF treatment had the highest probability for achieving HBV DNA undetectable after 48-week treatment; (b) network plot. The node size indicates the sample size in the treatment group that the node stands for; the thickness of the link represents the sample size of the direct comparisons.

After 48-week treatment (Figure 2(a)), no significant difference in the rate of virological breakthrough and genotypic resistance was observed when comparing TDF to LAM/ADV (0 versus 6.5%; OR, 0.21; 95% CI: 0.01–4.50). However, ETV had higher rate of virological breakthrough and genotypic resistance compared to LAM/ADV (12.4% versus 0.79%; OR, 5.84; 95% CI: 1.15–29.78).

After 96-week treatment (Figure 2(b)), there was no significant difference in virological breakthrough and genotypic resistance between ETV and ADV (25% versus 13.6%; OR, 2.11; 95% CI: 0.60–7.46). However, ETV (22.9% versus 4.2%; OR, 5.98; 95% CI: 1.70–21.10) or ADV (26% versus 10.7%; OR, 3.22; 95% CI: 1.37–7.55) had higher rate of virological breakthrough and genotypic resistance compared to LAM/ADV, respectively.

#### 3.5.2. Network Meta-Analysis

On Bayesian network meta-analysis, ETV had higher virological breakthrough and genotypic resistance as compared to TDF (OR, 31.37; 95% CrI: 1.62–605.81) or LAM/ADV (OR, 6.49; 95% CrI: 1.48–28.54) through 48 weeks of treatment (Sup. Table 4).

### 3.6. Sensitivity Analysis

Additionally, in direct meta-analysis, we only found there was clinical heterogeneity in 24-week (*I* ^2^ = 75%) and 48-week (*I* ^2^ = 59%) undetectable HBV DNA rate between ETV group and LAM plus ADV group. In 24-week virological response, the heterogeneity was brought by Maklad et al. 2014 study [18]; ETV had lower rate of virological response compared to LAM/ADV (OR, 0.29; 95% CI: 0.12–0.68; *P* = 0.004; *I* ^2^ = 0.0%; Sup. Figure 7(A)) after removing this study. In 48-week virological response, the heterogeneity was brought by Ong et al.'s 2011 study [16]. Because we did network meta-analysis of 48 weeks' virological response, heterogeneity in 48 weeks' virological response should be discussed to find its influence on the synthetic results of network meta-analysis.

When the heterogeneity was removed by excluding one study [16], there was still no significant difference in the direct meta-analysis of 48 weeks' virological response between ETV and LAM/ADV (OR, 0.63; 95% CI: 0.38–1.03; *P* = 0.064; *I* ^2^ = 3.3%; Sup. Figure 7(B)). In the network meta-analysis, TDF still had higher rates of HBV DNA undetectable as compared to ETV (OR, 25.58; 95% CrI: 4.67–140.24), ADV (OR, 42.43; 95% CrI: 11.25–160.06), or LAM/ADV (OR, 16.55; 95% CrI: 3.74–73.29) through 48 weeks of treatment. No drug was superior to others among ETV, ADV, and LAM/ADV. Also no change was made in rank probability of 48 weeks' HBV DNA undetectable rate. Therefore, even though heterogeneity was found in the direct meta-analysis of 48 weeks' virological response between ETV and LAM/ADV, it left no statistical influence on the synthetic results of network meta-analysis.

## 4. Discussion

This Bayesian network meta-analysis is the first study to evaluate the relative efficacy of TDF, ETV, LAM plus ADV, and ADV compared with each other for patients with LAM-R, in terms of rates of undetectable HBV DNA, ALT normalization, HBeAg loss, and virological breakthrough.

In direct meta-analysis, TDF showed a stronger antiviral effect than any one of ETV, LAM/ADV, and ADV against LAM-R hepatitis B virus. When comparing TDF to ETV, LAM/ADV, or ADV, the proportion of patients with serum HBV DNA levels < 400 copies/mL was 93.5% versus 14.8%, 88.9% versus 36.4%, or 86.4% versus 21.5% at 48 weeks, respectively. Meanwhile, LAM/ADV combination therapy was superior to ADV in suppressing viral replication for patients with LAM-R, and the proportion of patients with undetectable HBV DNA was 63.1% versus 30.2%, 75.0% versus 42.7%, and 83.3% versus 50.0% when comparing LAM/ADV to ADV at 24, 48, and 96 weeks, respectively. However, ETV had similar rates of HBV DNA undetectable compared to ADV or LAM/ADV. The rates of HBV DNA undetectable for ETV were 21.6% versus 40.7%, 32.3% versus 34.2%, and 48.6% versus 41.2% when comparing LAM/ADV through 24, 48, and 96 weeks' therapy, respectively. The rates of undetectable HBV DNA were 33.3% versus 27.3% and 54.2% versus 40.9% when comparing ETV to ADV at 24, 48, weeks, respectively. In the network meta-analysis, the results were basically similar to the results of the direct meta-analysis. Of note, on comparative effectiveness network meta-analysis among TDF, ETV, LAM/ADV, and ADV for patients with LAM-R by undetectable HBV DNA rate at 48 weeks, TDF and LAM/ADV had the highest probability of ranking first and second, respectively, whereas ETV and ADV had highest probability of being ranked third and fourth, respectively.

ALT normalization and HBeAg loss are usually associated with improved clinical outcomes. In this study, among TDF, ETV, LAM/ADV, and ADV, the results of direct and indirect meta-analysis revealed that no drug was clearly superior to others in the rates of ALT normalization and HBeAg loss compared with each other after 48 weeks of therapy. However, on comparative effectiveness network meta-analysis, TDF, ADV, and LAM/ADV had the highest probability of being ranked first, second, and third for improving biochemical response, respectively, whereas ETV had the worst probability of being ranked fourth. In addition, TDF, LAM/ADV, ADV, and ETV had the highest probability of being ranked first, second, third, and fourth for achieving HBeAg loss after 48-week treatment, respectively. Of note, ETV or ADV had a higher rate of virological breakthrough and genotypic resistance compared to LAM/ADV (22.9% versus 4.2%) or (26% versus 10.7%) through 96-week therapy; however, no patient included in our meta-analysis had virological breakthrough during TDF treatment.

TDF and ETV are highly potent antivirals with high genetic barriers in NA-naïve patients [27, 28]; however, direct evidence in this study demonstrated superiority of TDF over ETV for suppression of HBV DNA replication in patients with LAM-R. Although the finding was based on a single trial, our network meta-analysis by comparing TDF versus ETV, LAM/ADV versus ETV, and ADV versus ETV provided a comprehensive efficacy of ETV in patients with ADV-R, which revealed that ETV was less effective in LAM-R patients compared to TDF or LAM/ADV. Meanwhile, this study provided a comparison of the antiviral efficacy between TDF and LAM-ADV or ADV for patients with LAM-R and showed that TDF alone exerted greater viral suppression than LAM plus ADV or ADV alone therapy for these patients. Therefore, TDF mono-rescue therapy is superior to not only ETV or ADV alone but also the combination of LAM and ADV therapy in effectively suppressed viral activity in patients with LAM-R. In addition, our results showed that LAM plus ADV therapy for up to 96 weeks achieved better rates of virological suppression and resistance than ADV alone, which was consistent with previous meta-analyses by Chen et al. [25]. Although ETV alone had similar efficacy in viral suppression compared to LAM plus ADV therapy through 96 week of treatment, ETV-treated patients showed relatively high probabilities of virological breakthrough and resistance mutation compared to those of LAM plus ADV therapy. Since preventing the development of multidrug resistance is a key concern for evaluating the efficacy of rescue therapy for these patients with genotypic resistance [4, 5], therefore, taking into account all outcomes in the study, our results suggest that TDF appears to be a more effective rescue therapy than LAM/ADV, ETV, or ADV for patients with LAM-R, and ETV or ADV alone is not a reasonable therapeutic option for these patients. In addition, our network meta-analysis also presented the probabilities of ranking for all these treatment strategies by using the Bayesian approach. The results of the probabilities of ranking could help the clinicians to choose better decisions for treatment.

Several limitations regarding of our systematic review require comment. Firstly, some studies had a small sample size and some of the reports' experimental controls were not very balanced. Secondly, it has been reported that geographic, ethnic, or disease status (such as baseline disease characteristics, genotype, pervious treatment history, etc.) differences are possibly associated with agent efficacy. Thirdly, long-term outcomes of TDF in treatment of LAM-R patients were not adequately assessed owing to limited published studies in this area.

In conclusion, TDF monotherapy appears to be a more effective rescue therapy than LAM/ADV, ETV, or ADV for patients with LAM-R. LAM and ADV combination therapy was a better treatment option than ETV or ADV alone. ETV or ADV monotherapy is not a reasonable therapeutic option for CHB patients with LAM-R.

## Supplementary Material

Supplementary material 1: Supplementary Table 1. 48 weeks of network meta-analysis of undetectable HBV DNA rate. Supplementary Table 2. 48 weeks of network meta-analysis of ALT normalization rate. Supplementary Table 3. 48 weeks of network meta-analysis of HBeAg loss rate. Supplementary Table 4. 48 weeks of network meta-analysis of Viral breakthrough and genotypic. Supplementary Figure 1. Flow diagram. Flow diagram of the studies identified. Supplementary Figure 2. Assessment of risk of bias. (A) Summary of risk of bias for each trail assessed, plus sign was for a judgment of Yes or low risk of bias, minus sign was for a judgment of No or high risk of bias, and question mark was for a judgment of Unclear, or uncertain risk of bias; (B) risk of bias graph about each risk of bias item presented as percentages across all included studies. Supplemental Figure 3. Forest plot of direct meta-analysis of 48 weeks of ALT normalization rate. Supplemental Figure 4. Forest plot of direct meta-analysis of HBeAg clearance rate. (A) 48 weeks; (B) 96 weeks. Supplement Figure 5. Rank probability and network plot of 48 weeks of ALT normalization rate.(A) rank probability; (B) network plot. Supplement Figure 6. Rank probability and network plot of 48 weeks of HBeAg loss rate. (A) rank probability; (B) network plot. Supplement Figure 7. Forest plot of direct meta-analysis of virological response after removing studies brought heterogeneities. (A) 24 weeks; (B) 48 weeks.
Supplementary material 2: Newcastle-ottawa Scale for non-random studies.

## Figures and Tables

**Table 1:** Characteristics of the included studies.

Author (year)	Interventions	Group (*n*)^*∗*^	Country	Age (y)	Baseline HBV DNA level (log10 IU/mL)	Baseline ALT level (U/L)	HBeAg+ (%)	Study design
Hann et al. [15] (2008)	TDF (NA) ADV (NA)	TDF (44) ADV (65)	USA	46 (11)	6.4 (1.6)	91 (161)	78	nRCT

Ong et al. [16] (2011)	TDF (NA) ETV (NA)	TDF (18) ETV (11) LAM/ADV (77)	Hong Kong, China	48.1 ± 11.4	5.78 ± 1.67	89 (44–210)	50	nRCT

Yang et al. [17] (2015)	TDF^$^ LAM/ADV^#^	TDF (28) LAM/ADV (31)	China	36.36 ± 10.14	5.08 ± 1.11	101.54 ± 26.14	88.14	RCT

Maklad et al. [18] (2014)	LAM/ADV^#^ ETV^&^	LAM/ADV (25) ETV (13)	Egypt	38.5 ± 11.5	5.57 ± 4.41	69.75 ± 31.5	41.4	nRCT

Ze et al. [19] (2014)	LAM/ADV^#^ ETV^&^	LAM/ADV (36) ETV (24)	Korea	52.5 ± 11	7.1 ± 1.5	195 ± 188	91	nRCT

Chung et al. [20] (2011)	LAM/ADV^#^ ETV^&^	LAM/ADV (52) ETV (44)	Korea	51.85 ± 10.45	6.85 ± 1.1	172 ± 155	63.45	nRCT

Ryu et al. [21] (2010)	LAM/ADV^#^ ETV^&^	LAM/ADV (47) ETV (45)	Korea	44 (21–64)	7.36 (5.31–9.48)	122 (22–887)	88.15	RCT

Kim et al. [22] (2010)	LAM/ADV^#^ ADV^@^ ETV^&^	LAM/ADV (36) ADV (44) ETV (24)	Korea	45.3 ± 9.57	6.69 ± 1.46	165.37 ± 185.5	69.3	nRCT

Wang et al. [23] (2014)	LAM/ADV^#^ ADV^@^	LAM/ADV (48) ADV (52)	China	42.8 ± 10.15	5.1 ± 1.12	100.3 ± 38.45	84.05	nRCT

^*∗*^CHB patients with LAM-R treated with tenofovir disoproxil fumarate (TDF), lamivudine plus adefovir (LAM/ADV), entecavir (ETV), or adefovir (ADV), respectively.

TDF^$^: patients treated with 300 g/d TDF; LAM/ADV^#^: patients treated with 100 g/d LAM and 10 mg/d ADV; ETV^&^: patients treated with 1 mg/d ETV; ADV^@^: patients treated with 10 mg/d ADV; NA: data not available.
