# Supplementary material for: Antiviral Therapy in Lamivudine-Resistant Chronic Hepatitis B Patients: A Systematic Review and Network Meta-Analysis
Source: Gastroenterol Res Pract. 2016 Sep 8;2016:3435965. doi: 10.1155/2016/3435965 (PMC5031861; doi:10.1155/2016/3435965)
Supplement: Supplementary file 1 — Supplementary material 1: Supplementary Table 1. 48 weeks of network meta-analysis of undetectable HBV DNA rate. Supplementary Table 2. 48 weeks of network meta-analysis of ALT normalization rate. Supplementary Table 3. 48 weeks of network meta-analysis of HBeAg loss rate. Supplementary Table 4. 48 weeks of network meta-analysis of Viral breakthrough and genotypic. Supplementary Figure 1. Flow diagram. Flow diagram of the studies identified. Supplementary Figure 2. Assessment of risk of bias. (A) Summary of risk of bias for each trail assessed, plus sign was for a judgment of Yes or low risk of bias, minus sign was for a judgment of No or high risk of bias, and question mark was for a judgment of Unclear, or uncertain risk of bias; (B) risk of bias graph about each risk of bias item presented as percentages across all included studies. Supplemental Figure 3. Forest plot of direct meta-analysis of 48 weeks of ALT normalization rate. Supplemental Figure 4. Forest plot of direct meta-analysis of HBeAg clearance rate. (A) 48 weeks; (B) 96 weeks. Supplement Figure 5. Rank probability and network plot of 48 weeks of ALT normalization rate.(A) rank probability; (B) network plot. Supplement Figure 6. Rank probability and network plot of 48 weeks of HBeAg loss rate. (A) rank probability; (B) network plot. Supplement Figure 7. Forest plot of direct meta-analysis of virological response after removing studies brought heterogeneities. (A) 24 weeks; (B) 48 weeks. Supplementary material 2: Newcastle-ottawa Scale for non-random studies. [file 3435965.f1.docx]

**Supplemental material 1:**

**Supplementary Figure 1 Flow diagram.** Flow diagram of the studies identified.

**Supplementary Figure 2 Assessment of risk of bias.** (A) Summary of risk of bias for each trail assessed, plus sign was for a judgment of Yes or low risk of bias, minus sign was for a judgment of No or high risk of bias, and question mark was for a judgment of Unclear, or uncertain risk of bias; (B) risk of bias graph about each risk of bias item presented as percentages across all included studies.

**Supplemental Figure 3 Forest plot of direct meta-analysis of 48 weeks of ALT normalization rate.**

**Supplemental Figure 4 Forest plot of direct meta-analysis of HBeAg clearance rate.** (A) 48 weeks; (B) 96 weeks.

**Supplement Figure 5 Rank probability and network plot of 48 weeks of ALT normalization rate.**(A) rank probability; (B) network plot.

**Supplement Figure 6 Rank probability and network plot of 48 weeks of HBeAg loss rate.** (A) rank probability; (B) network plot.

**Supplement Figure 7 Forest plot of direct meta-analysis of virological response after removing studies brought heterogeneities. (**A) 24 weeks; (B) 48 weeks.

Supplemental Table1 48 weeks of network meta-analysis of undetectable HBV DNA rate

|  | TDF-LAM-R | ETV-LAM-R | ADV-LAM-R | (LAM-ADV)-LAM-R |
| --- | --- | --- | --- | --- |
| TDF-LAM-R |  | 24.686(5.362- 113.659) | 37.281(9.725- 142.921) | 21.053(5.697- 77.801) |
| ETV-LAM-R | 0.404(0.012- 13.557) |  | 1.235(0.326- 4.678) | 0.724(0.248- 2.111) |
| ADV-LAM-R | 0.069(0.002- 1.932) | 0.546(0.151- 1.975) |  | 0.431(0.148- 1.251) |
| (LAM-ADV)-LAM-R | 0.141(0.005- 4.092) | 1.275(0.411- 3.957) | 1.854(0.570- 6.035) |  |

Supplement Table 2 48 weeks of network meta-analysis of ALT normalization rate

|  | TDF-LAM-R | ETV-LAM-R | ADV-LAM-R | (LAM-ADV)-LAM-R |
| --- | --- | --- | --- | --- |
| TDF-LAM-R |  | 2.717(0.120- 61.571) | 1.361(0.170- 10.917) | 1.872(0.233- 15.020) |
| ETV-LAM-R | 0.368(0.016- 8.341) |  | 0.501(0.022- 11.350) | 0.689(0.067- 7.04) |
| ADV-LAM-R | 0.735(0.092- 5.896) | 1.997(0.088- 45.249) |  | 1.376(0.171- 11.038) |
| (LAM-ADV)-LAM-R | 0.534(0.067- 4.285) | 1.451(0.142- 14.832) | 0.727(0.091- 5.831) |  |

Supplement Table 3 48 weeks of network meta-analysis of HBeAg loss rate

|  | TDF-LAM-R | ETV-LAM-R | ADV-LAM-R | (LAM-ADV)-LAM-R |
| --- | --- | --- | --- | --- |
| TDF-LAM-R |  | 5.343(0.826- 34.547) | 3.145(0.969- 10.205) | 2.204(0.668- 7.268) |
| ETV-LAM-R | 0.187(0.029- 1.210) |  | 0.589(0.092- 3.757) | 0.412(0.098- 1.732) |
| ADV-LAM-R | 0.318(0.098- 1.032) | 1.699(0.266- 10.842) |  | 0.701(0.217- 2.264) |
| (LAM-ADV)-LAM-R | 0.454(0.138- 1.497) | 2.425(0.577- 10.185) | 1.427(0.442- 4.612) |  |

Supplement Table 4 48 weeks of network meta-analysis of Viral breakthrough and genotypic resistance

|  | TDF-LAM-R | ETV-LAM-R | (LAM-ADV)-LAM-R |
| --- | --- | --- | --- |
| TDF-LAM-R |  | 0.032(0.002 -0.616) | 0.207(0.016 -2.689) |
| ETV-LAM-R | 31.369(1.624 -605.812) |  | 6.494(1.478 -28.538) |
| (LAM-ADV)-LAM-R | 4.831(0.372  -62.743) | 0.154(0.035 -0.677) |  |


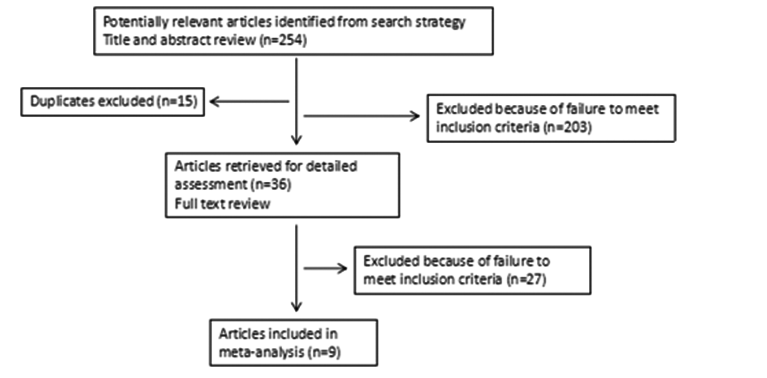


Supplementary Figure 1


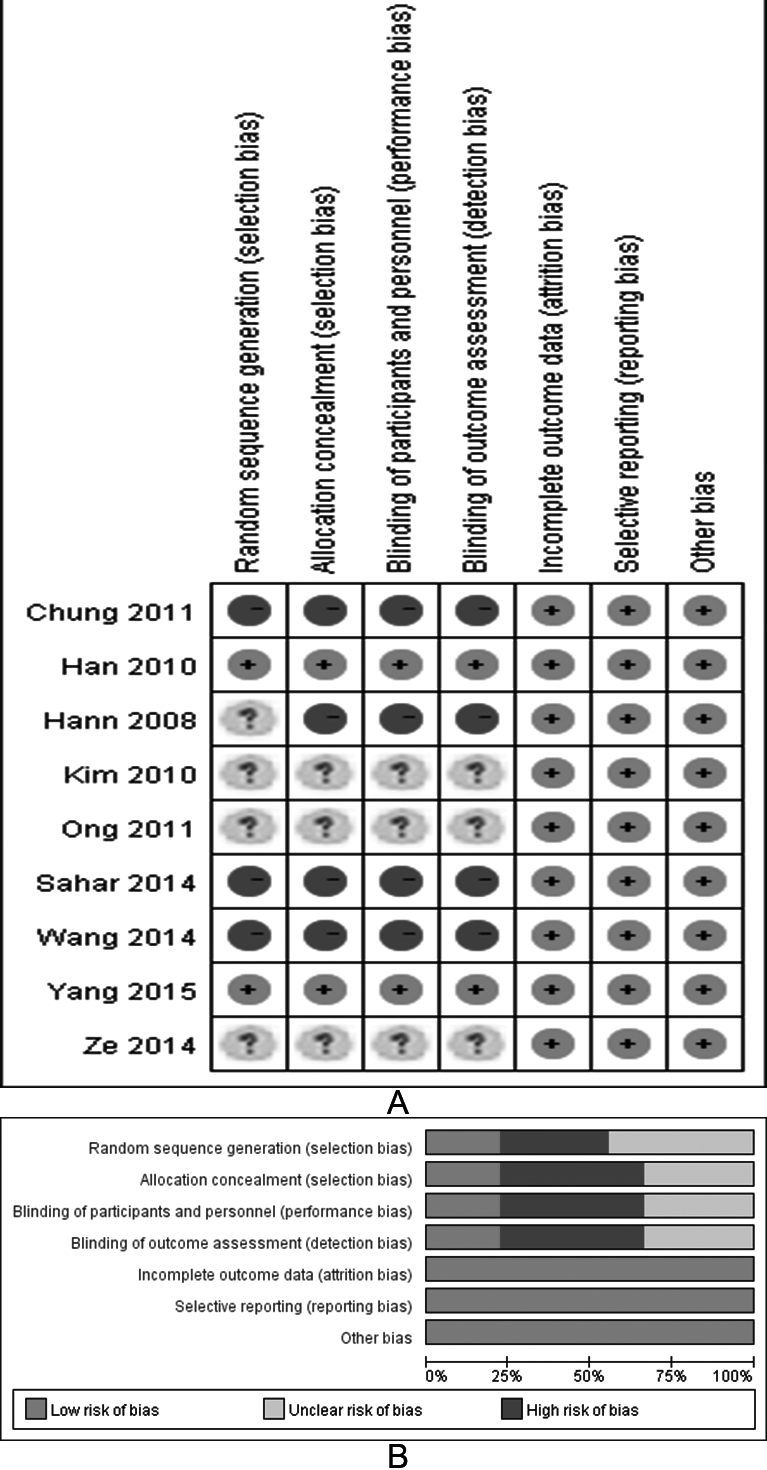


Supplementary Figure 2


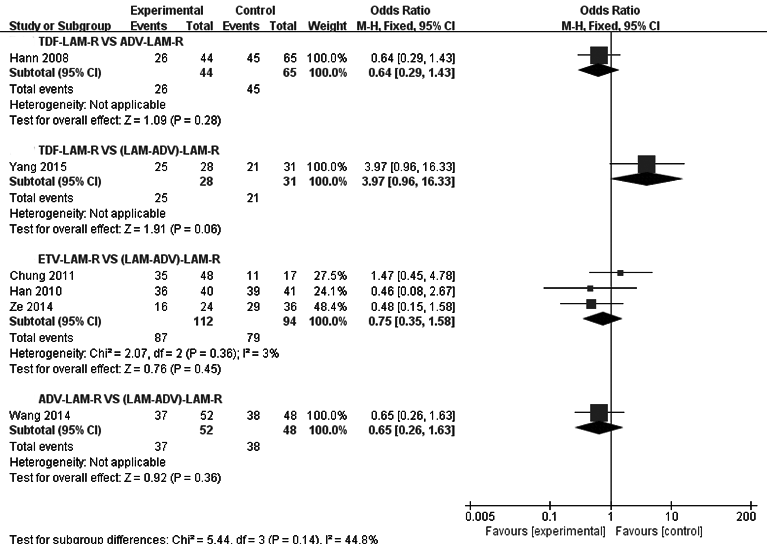


Supplementary Figure 3


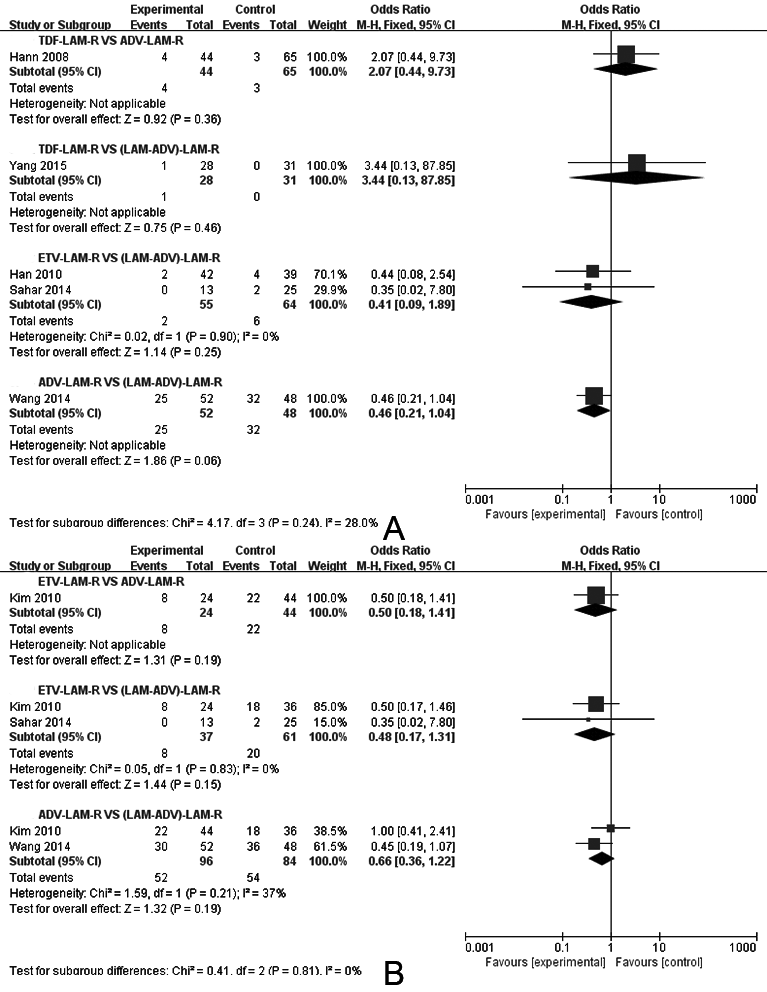


Supplementary Figure 4


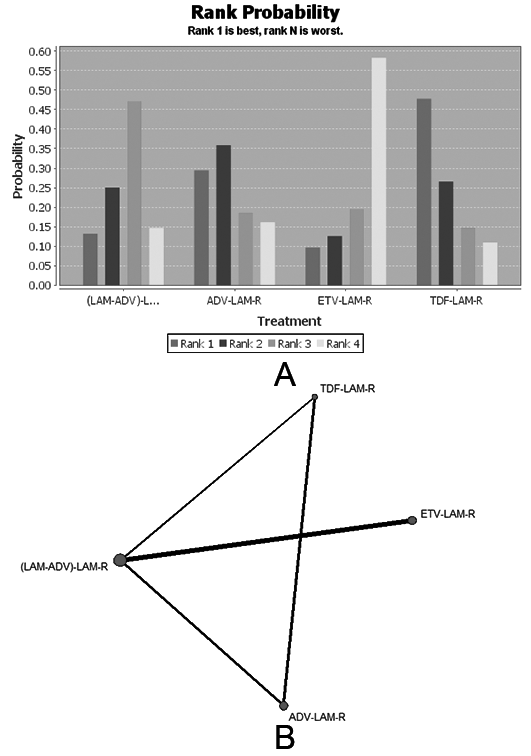


Supplementary Figure 5


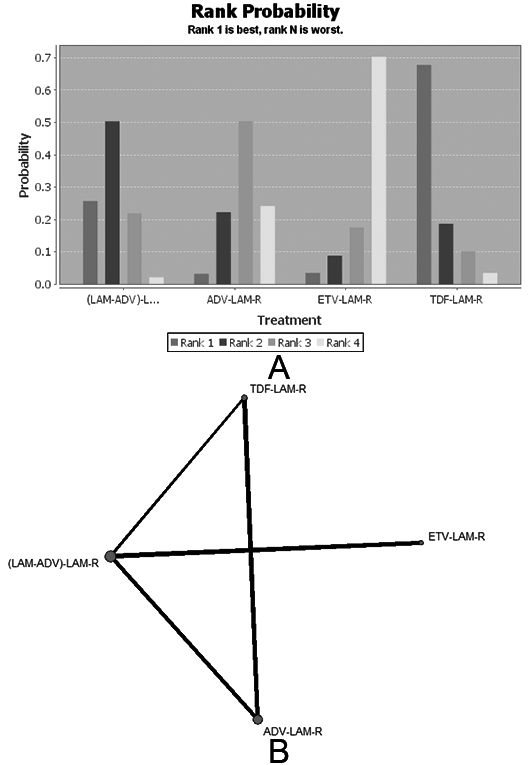


Supplementary Figure 6


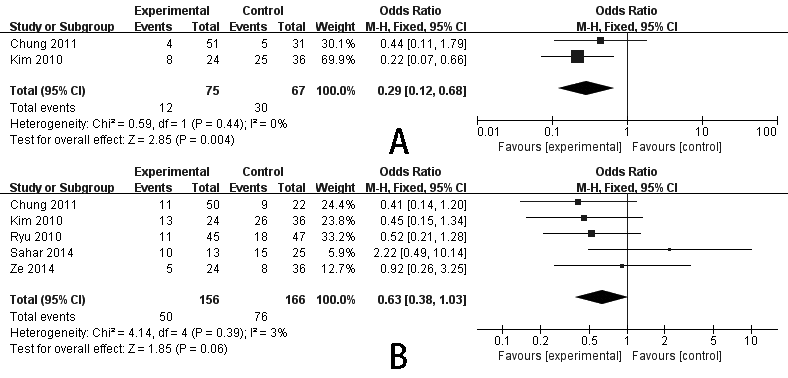


Supplementary Figure 7

**Supplemental material 2-Newcastle-ottawa Scale for non-random studies:**

Note: A study can be awarded a maximum of one star for each numbered item within the Selection and Outcome categories. A maximum of two stars can be given for Comparability

**COHORT STUDIES (Hann 2008)**

**Selection**

1) Representativeness of the exposed cohort

a) truly representative of the average ______ LAM resistance_______ (describe) in the community **🟑**

b) somewhat representative of the average ______________ in the community **🟑**

c) selected group of users eg nurses, volunteers

d) no description of the derivation of the cohort

2) Selection of the non exposed cohort

a) drawn from the same community as the exposed cohort **🟑 √**

b) drawn from a different source

c) no description of the derivation of the non exposed cohort

3) Ascertainment of exposure

a) secure record (eg surgical records) **🟑 √**

b) structured interview **🟑**

c) written self report

d) no description

4) Demonstration that outcome of interest was not present at start of study

a) yes **🟑 √**

b) no

**Comparability**

1) Comparability of cohorts on the basis of the design or analysis

a) study controls for _____________ (select the most important factor) **🟑**

b) study controls for any additional factor **🟑** (This criteria could be modified to indicate specific control for a second important factor.)

**Outcome**

1) Assessment of outcome

a) independent blind assessment **🟑** **√**

b) record linkage **🟑**

c) self report

d) no description

2) Was follow-up long enough for outcomes to occur

a) yes (select an adequate follow up period for outcome of interest) **🟑√**

b) no

3) Adequacy of follow up of cohorts

a) complete follow up - all subjects accounted for **🟑** **√**

b) subjects lost to follow up unlikely to introduce bias - small number lost - > ____ % (select an adequate %) follow up, or description provided of those lost) **🟑**

c) follow up rate < ____% (select an adequate %) and no description of those lost

d) no statement

**COHORT STUDIES (Ong 2011)**

**Selection**

1) Representativeness of the exposed cohort

a) truly representative of the average _ chronic hepatitis B patients withHBsAg (describe) in the community **🟑**

b) somewhat representative of the average ______________ in the community **🟑**

c) selected group of users eg nurses, volunteers

d) no description of the derivation of the cohort

2) Selection of the non exposed cohort

a) drawn from the same community as the exposed cohort **🟑 √**

b) drawn from a different source

c) no description of the derivation of the non exposed cohort

3) Ascertainment of exposure

a) secure record (eg surgical records) **🟑 √**

b) structured interview **🟑**

c) written self report

d) no description

4) Demonstration that outcome of interest was not present at start of study

a) yes **🟑 √**

b) no

**Comparability**

1) Comparability of cohorts on the basis of the design or analysis

a) study controls for _____________ (select the most important factor) **🟑**

b) study controls for any additional factor **🟑** (This criteria could be modified to indicate specific control for a second important factor.)

**Outcome**

1) Assessment of outcome

a) independent blind assessment **🟑** **√**

b) record linkage **🟑**

c) self report

d) no description

2) Was follow-up long enough for outcomes to occur

a) yes (select an adequate follow up period for outcome of interest) **🟑√**

b) no

3) Adequacy of follow up of cohorts

a) complete follow up - all subjects accounted for **🟑** **√**

b) subjects lost to follow up unlikely to introduce bias - small number lost - > ____ % (select an adequate %) follow up, or description provided of those lost) **🟑**

c) follow up rate < ____% (select an adequate %) and no description of those lost

d) no statement

**COHORT STUDIES (Sahar Maklad 2014)**

**Selection**

1) Representativeness of the exposed cohort

a) truly representative of the average _ lamivudine-resistant (describe) in the community **🟑**

b) somewhat representative of the average ______________ in the community **🟑**

c) selected group of users eg nurses, volunteers

d) no description of the derivation of the cohort

2) Selection of the non exposed cohort

a) drawn from the same community as the exposed cohort **🟑 √**

b) drawn from a different source

c) no description of the derivation of the non exposed cohort

3) Ascertainment of exposure

a) secure record (eg surgical records) **🟑 √**

b) structured interview **🟑**

c) written self report

d) no description

4) Demonstration that outcome of interest was not present at start of study

a) yes **🟑 √**

b) no

**Comparability**

1) Comparability of cohorts on the basis of the design or analysis

a) study controls for ___ HBeAg-positive__ (select the most important factor) **🟑**

b) study controls for any additional factor **🟑** (This criteria could be modified to indicate specific control for a second important factor.)

**Outcome**

1) Assessment of outcome

a) independent blind assessment **🟑** **√**

b) record linkage **🟑**

c) self report

d) no description

2) Was follow-up long enough for outcomes to occur

a) yes (select an adequate follow up period for outcome of interest) **🟑√**

b) no

3) Adequacy of follow up of cohorts

a) complete follow up - all subjects accounted for **🟑** **√**

b) subjects lost to follow up unlikely to introduce bias - small number lost - > ____ % (select an adequate %) follow up, or description provided of those lost) **🟑**

c) follow up rate < ____% (select an adequate %) and no description of those lost

d) no statement

**COHORT STUDIES (Ze 2014)**

**Selection**

1) Representativeness of the exposed cohort

a) truly representative of the average ______ LAM resistance_______ (describe) in the community **🟑**

b) somewhat representative of the average ______________ in the community **🟑**

c) selected group of users eg nurses, volunteers

d) no description of the derivation of the cohort

2) Selection of the non exposed cohort

a) drawn from the same community as the exposed cohort **🟑 √**

b) drawn from a different source

c) no description of the derivation of the non exposed cohort

3) Ascertainment of exposure

a) secure record (eg surgical records) **🟑 √**

b) structured interview **🟑**

c) written self report

d) no description

4) Demonstration that outcome of interest was not present at start of study

a) yes **🟑 √**

b) no

**Comparability**

1) Comparability of cohorts on the basis of the design or analysis

a) study controls for _____________ (select the most important factor) **🟑**

b) study controls for any additional factor **🟑** (This criteria could be modified to indicate specific control for a second important factor.)

**Outcome**

1) Assessment of outcome

a) independent blind assessment **🟑** **√**

b) record linkage **🟑**

c) self report

d) no description

2) Was follow-up long enough for outcomes to occur

a) yes (select an adequate follow up period for outcome of interest) **🟑√**

b) no

3) Adequacy of follow up of cohorts

a) complete follow up - all subjects accounted for **🟑** **√**

b) subjects lost to follow up unlikely to introduce bias - small number lost - > ____ % (select an adequate %) follow up, or description provided of those lost) **🟑**

c) follow up rate < ____% (select an adequate %) and no description of those lost

d) no statement

**COHORT STUDIES (Chung 2011)**

**Selection**

1) Representativeness of the exposed cohort

a) truly representative of the average __ LAM-resistant____ (describe) in the community **🟑**

b) somewhat representative of the average ______________ in the community **🟑**

c) selected group of users eg nurses, volunteers

d) no description of the derivation of the cohort

2) Selection of the non exposed cohort

a) drawn from the same community as the exposed cohort **🟑 √**

b) drawn from a different source

c) no description of the derivation of the non exposed cohort

3) Ascertainment of exposure

a) secure record (eg surgical records) **🟑 √**

b) structured interview **🟑**

c) written self report

d) no description

4) Demonstration that outcome of interest was not present at start of study

a) yes **🟑 √**

b) no

**Comparability**

1) Comparability of cohorts on the basis of the design or analysis

a) study controls for _____________ (select the most important factor) **🟑**

b) study controls for any additional factor **🟑** (This criteria could be modified to indicate specific control for a second important factor.)

**Outcome**

1) Assessment of outcome

a) independent blind assessment **🟑** **√**

b) record linkage **🟑**

c) self report

d) no description

2) Was follow-up long enough for outcomes to occur

a) yes (select an adequate follow up period for outcome of interest) **🟑√**

b) no

3) Adequacy of follow up of cohorts

a) complete follow up - all subjects accounted for **🟑** **√**

b) subjects lost to follow up unlikely to introduce bias - small number lost - > ____ % (select an adequate %) follow up, or description provided of those lost) **🟑**

c) follow up rate < ____% (select an adequate %) and no description of those lost

d) no statement

**COHORT STUDIES (Kim 2010)**

**Selection**

1) Representativeness of the exposed cohort

a) truly representative of the average __LAM-resistant__ (describe) in the community **🟑**

b) somewhat representative of the average ______________ in the community **🟑**

c) selected group of users eg nurses, volunteers

d) no description of the derivation of the cohort

2) Selection of the non exposed cohort

a) drawn from the same community as the exposed cohort **🟑 √**

b) drawn from a different source

c) no description of the derivation of the non exposed cohort

3) Ascertainment of exposure

a) secure record (eg surgical records) **🟑 √**

b) structured interview **🟑**

c) written self report

d) no description

4) Demonstration that outcome of interest was not present at start of study

a) yes **🟑 √**

b) no

**Comparability**

1) Comparability of cohorts on the basis of the design or analysis

a) study controls for _____________ (select the most important factor) **🟑**

b) study controls for any additional factor **🟑** (This criteria could be modified to indicate specific control for a second important factor.)

**Outcome**

1) Assessment of outcome

a) independent blind assessment **🟑** **√**

b) record linkage **🟑**

c) self report

d) no description

2) Was follow-up long enough for outcomes to occur

a) yes (select an adequate follow up period for outcome of interest) **🟑√**

b) no

3) Adequacy of follow up of cohorts

a) complete follow up - all subjects accounted for **🟑** **√**

b) subjects lost to follow up unlikely to introduce bias - small number lost - > ____ % (select an adequate %) follow up, or description provided of those lost) **🟑**

c) follow up rate < ____% (select an adequate %) and no description of those lost

d) no statement

**COHORT STUDIES (Wang 2014)**

**Selection**

1) Representativeness of the exposed cohort

a) truly representative of the average __ LAM-resistance_ (describe) in the community **🟑**

b) somewhat representative of the average ______________ in the community **🟑**

c) selected group of users eg nurses, volunteers

d) no description of the derivation of the cohort

2) Selection of the non exposed cohort

a) drawn from the same community as the exposed cohort **🟑 √**

b) drawn from a different source

c) no description of the derivation of the non exposed cohort

3) Ascertainment of exposure

a) secure record (eg surgical records) **🟑 √**

b) structured interview **🟑**

c) written self report

d) no description

4) Demonstration that outcome of interest was not present at start of study

a) yes **🟑 √**

b) no

**Comparability**

1) Comparability of cohorts on the basis of the design or analysis

a) study controls for _____________ (select the most important factor) **🟑**

b) study controls for any additional factor **🟑** (This criteria could be modified to indicate specific control for a second important factor.)

**Outcome**

1) Assessment of outcome

a) independent blind assessment **🟑** **√**

b) record linkage **🟑**

c) self report

d) no description

2) Was follow-up long enough for outcomes to occur

a) yes (select an adequate follow up period for outcome of interest) **🟑√**

b) no

3) Adequacy of follow up of cohorts

a) complete follow up - all subjects accounted for **🟑** **√**

b) subjects lost to follow up unlikely to introduce bias - small number lost - > ____ % (select an adequate %) follow up, or description provided of those lost) **🟑**

c) follow up rate < ____% (select an adequate %) and no description of those lost

d) no statement
